# Role of p90RSK in Kidney and Other Diseases

**DOI:** 10.3390/ijms20040972

**Published:** 2019-02-23

**Authors:** Ling Lin, Samantha A. White, Kebin Hu

**Affiliations:** Department of Cellular and Molecular Physiology, The Pennsylvania State University College of Medicine, 500 University Drive, Hershey, PA 17033, USA; swhite11@pennstatehealth.psu.edu

**Keywords:** p90RSK, signalling, kidney, atherosclerosis, kinase inhibitors, pathophysiology

## Abstract

The 90 kDa ribosomal s6 kinases (RSKs) are a group of serine/threonine kinases consisting of 4 RSK isoforms (RSK1-4), of which RSK1 is also designated as p90RSK. p90RSK plays an important role in the Ras-mitogen-activated protein kinase (MAPK) signalling cascade and is the direct downstream effector of Ras-extracellular signal-regulated kinase (ERK1/2) signalling. ERK1/2 activation directly phosphorylates and activates p90RSK, which, in turn, activates various signalling events through selection of different phosphorylation substrates. Upregulation of p90RSK has been reported in numerous human diseases. p90RSK plays an important role in the regulation of diverse cellular processes. Thus, aberrant activation of p90RSK plays a critical role in the pathogenesis of organ dysfunction and damage. In this review, we focus on the current understanding of p90RSK functions and roles in the development and progression of kidney diseases. Roles of p90RSK, as well as other RSKs, in cardiovascular disorders and cancers are also discussed.

## 1. Introduction

**Figure 1:**
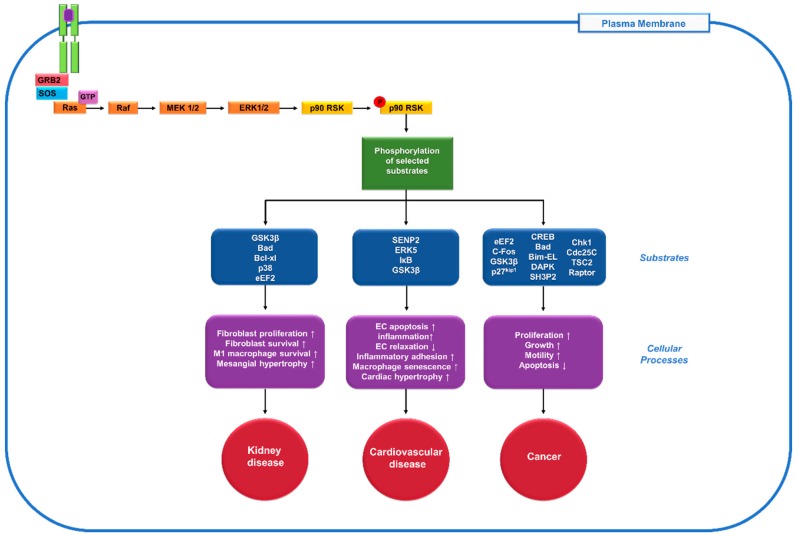
Role of the 90 kDa ribosomal s6 kinases (p90RSK), one of the downstream mediators of extracellular signal-regulated kinase (ERK) pathway, in diseases. When the receptor tyrosine kinase (RTK, green) gets activated by a ligand (purple), it stimulates the activation of the docking proteins growth factor receptor-bound protein 2 (GRB2) and son of sevenless (SOS). SOS helps remove the guanosine diphosphate (GDP) from Rat sarcoma (Ras), allowing it to bind guanosine triphosphate (GTP) and become activated. The Ras signalling cascade begins as Ras activates the Raf kinase, which phosphorylates mitogen-activated protein kinase kinase (MEK1/2), extracellular signal-regulated kinase 1/2 (ERK1/2) and p90RSK. Then p90RSK phosphorylates various downstream substrates (blue) to mediate diverse cellular processes (purple) and contribute to numerous human diseases including kidney disease, cardiovascular disease and cancer (red).

The Ras-mitogen-activated protein kinase (MAPK) pathway is an important regulator of diverse cellular processes [1,2]. The Ras-MAPK pathway is initiated by a ligand binding the receptor tyrosine kinase (RTK) receptor, followed by adaptor proteins, such as growth factor receptor-bound protein 2 (GRB2) and son of sevenless (SOS), docking onto the RTK and leading to activation of the associated Ras GTPases and recruitment of Raf. Raf then activates downstream MEK1/2 and extracellular signal-regulated kinase (ERK1/2). The 90 kDa ribosomal s6 kinases (RSKs) are a group of serine/threonine kinases that play important roles in the MAPK signalling cascade and are the direct downstream effectors of ERK1/2. ERK1/2 activation directly phosphorylates and activates RSKs [3,4], which, in turn, activate various signalling events through selection of different phosphorylation substrates and modulate diverse cellular processes, such as cell proliferation, survival and motility and so forth. Thus, aberrant activation of RSKs plays a critical role in the pathogenesis of organ dysfunction and damage (Figure 1).

## 2. Biology of RSKs

### 2.1. Discovery and Expression

First identified from *Xenopus* studies, the originally termed ribosomal S6 kinase was found to be responsible for phosphorylating ribosomal protein S6 [5]. Later, the protein was purified and renamed p90RSK based on its approximately 90 kDa weight [6]. The 90 kDa RSKs are a family of Serine/Threonine kinase proteins. This family consists of 4 isoforms in humans, termed RSK1, RSK2, RSK3 and RSK4, of which RSK1 is also designated as p90RSK. These kinase isoforms are highly homologous, with around 75% of the structure being identical. Additionally, the expression patterns are similar among RSK 1-3, with comparable levels of RSK1-3 detected in adult tissues including heart, brain, lung, kidney and pancreas [7]. RSK4 has the most diverse expression pattern, with past studies showing expression occurring during development and RSK4 deletions are common in x-linked mental retardation [8].

### 2.2. Structure

The structure of RSKs is noteworthy because all the members contain two functionally diverse domains called the N terminal kinase domain (NTKD) and the C terminal kinase domain (CTKD). The NTKD is part of the kinase A, G and C (AGC) family, while the CTKD is part of the calcium calmodulin dependent kinase (CaMK) family. The function of the CTKD is to receive signals from ERK 1/2 to auto-phosphorylate RSK and is important to activate NTKD. Once NTKD is activated by CTKD, it goes on to phosphorylate downstream targets [9]. These domains are connected by a linker region that is approximately 100 amino acids large, containing regulatory elements [10]. Importantly, the RSK isoforms all contain an ERK1/2 docking domain [11]. This allows RSK activation by ERK1/2. Additionally, a nearby location is important for RSK autophosphorylation, which may play a role in ERK1/2 dissociation and RSK activity progression [12].

### 2.3. Activation

RSK activation is complex due to the multiple players and activation sites. All the human isoforms of RSK have four conserved phosphorylation sites: Ser^221^, Ser^363^, Ser^380^ and Thr^573^ [13]. The mechanism of activation of RSK depends on each phosphorylation site. Ser^221^ is located in the NTKD and is phosphorylated in response to phosphoinositide-dependent kinase-1 (PDK1), a constitutively active serine threonine kinase [14]. Ser^363^ and Ser^380^ are both located in the linker region between the kinase domains. Ser^363^ is activated by ERK 1/2 phosphorylation, while Ser^380^ is phosphorylated by CTKD [15]. Interestingly, it has been shown that Ser^380^, when phosphorylated, can serve as a docking point for PDK1, which in turn causes the activation of Ser^221^ [16]. Thr^573^ is within the CTKD and is also phosphorylated by ERK1/2 [17].

In addition to these mechanisms of activation, p38 MAPK and fibroblast growth factor receptor-3 (FGFR3) have been shown to influence RSK regulation. p38 MAPK has been shown to activate RSK in dendritic cells via MAPK-activated kinases M2 and M3, which activate CTKD [18]. FGFR3 has been shown to interact with RSK2 through tyrosine phosphorylation. This phosphorylation promotes ERK binding and causes RSK2 activation [19].

### 2.4. Downstream Substrates

RSKs regulate diverse cellular processes through phosphorylation of selected downstream substrates from a constantly growing list. Both p90RSK (i.e., RSK1) and RSK2 have been shown to promote cell proliferation and growth [20]. One such way that p90RSK impacts cell proliferation is through phosphorylating Max dimerization protein-1 (Mad1), which alleviates its suppression of Myc and resulting in increased proliferation [21]. p90RSK also regulates cell growth and protein synthesis through modulating mTOR pathway. Specifically, it influences mTOR by phosphorylating both tuberous sclerosis complex 2 (TSC2) and Raptor [22,23]. Moreover, p90RSK phosphorylates and inhibits glycogen synthase kinase (GSK)3β, causing the release of Cyclin D1 and cell proliferation [24] and inducing translation initiation factor eIF4B and protein synthesis [17,25]. RSKs have also been shown to interact with c-Fos, a transcription factor which is important during G1 phase of the cell cycle [26]; and phosphorylate p27^kip1^ to induce G1 phase progression [17,27]. Cell survival is also influenced by p90RSK, as it is known to phosphorylate Bad to decrease apoptosis and phosphorylate tumour suppressor death-associated protein kinase (DAPK) to cause its inactivation [28,29,30]. p90RSK has been shown to influence inflammation transcription through phosphorylating nuclear factor kappa-light-chain-enhancer of activated B cells (NF-κB) inhibitors, nuclear factor of kappa light polypeptide gene enhancer in B-cells inhibitor (IκB)α and IκBβ [31,32,33] and through phosphorylating p38 MAPK to induce M1 macrophage survival [34]. p90RSK also phosphorylates downstream substrates filamin A and phosphorylating SH3 domain-containing protein (SH3P2) to induce cell motility and migration [35,36].

## 3. p90RSK and Kidney Diseases

Increased p90RSK activation, that is, phosphorylation, is implicated in the aetiology of various diseases such as different types of cancers, cardiovascular diseases and kidney diseases [34,37]. In vitro, ERK and p90RSK have been shown to regulate multiple cellular processes including growth, cell proliferation, survival and motility through various downstream substrates in different cell types [38]. In vivo, cells with higher p90RSK expression and activity manifest abnormal growth and proliferation, more resistant to apoptosis and functional incapacity, causing dysfunction of the organs and progression of the diseases. Of the four RSKs, p90RSK is predominantly expressed in the kidney [1,3], suggesting that p90RSK may play a unique role in the pathogenesis of kidney diseases.

### 3.1. p90RSK and Kidney Fibrosis

In the obstruction-induced fibrotic kidney, the phosphorylation of p90RSK and its upstream signalling such as ERK1/2 is markedly induced after obstructive injury. The activation of p90RSK correlates with the severity of kidney fibrosis [34]. In vitro, profibrotic factors such as tissue-type plasminogen activator (tPA) and TGF-β can induce p90RSK phosphorylation and activation in kidney cells [30,34,39]. These implicate that p90RSK may involve in the initiation and progression of chronic kidney disease and renal fibrosis.

Interstitial fibroblasts and myofibroblasts are generally considered as the primary matrix-producing cells in kidney. The number of interstitial fibroblasts and myofibroblasts closely correlates with the severity of tubulointerstitial fibrosis and concomitant decline of kidney function [30]. Our recent work has shown that, in kidney fibroblasts, tPA binds to its receptor LRP-1 and induces its tyrosine phosphorylation, which, in turn, activates the downstream ERK1/2 and p90RSK pathways. The activation and phosphorylation of p90RSK then phosphorylates the downstream substrates, such as GSK-3β and Bad, to induce fibroblast proliferation and survival [24,30]. GSK-3β is constitutively active and inhibits cyclinD1 and other downstream mediators through ubiquitination and proteasomal degradation. Phosphorylation of GSK-3β inactivates the GSK-3β, resulting in cyclin D1 stabilization and accumulation, which facilitates fibroblasts entry into the S phase and induces cell proliferation [24]. Bad is a pro-death member of the Bcl-2 protein family. Phosphorylation of Bad inactivates it and prevents it from entering into the mitochondria after injury leading to suppression of cytochrome C releasing into the cytosol. Decreased cytosol cytochrome C reduces the cleavage and activation of the caspases, resulting in cell survival [30]. Thus, in tPA profibrotic signal pathway, p90RSK activation induces fibroblast accumulation and contributes to renal fibrosis through dual mechanisms: (1) to promote fibroblast survival through p90RSK/Bad/cytochrome C pathway; (2) to induce fibroblast proliferation through p90RSK/GSK-3β/cyclin D1 signalling [24,30].

### 3.2. p90RSK and Kidney Inflammation

Macrophage accumulation is one of the histological hallmarks of chronic kidney disease. In diseased conditions, macrophages are differentiated into two categories: M1 as classically activated and M2 as alternatively activated. After exposure to IFN-γ or lipopolysaccharide (LPS), macrophages (M1) not only produce nitric oxide or reactive oxygen intermediates to protect against bacteria or viral pathogens but also produce abundant proinflammatory cytokines to initiate immune response, recruit more inflammatory cells and exaggerate damage. After exposure to IL4, IL10 or IL13, macrophages (M2) produce polyamines and proline to help wound healing and tissue repair [34,40,41]. We discovered that p90RSK, phosphorylated by tPA, protects macrophage against apoptosis induced by hydrogen peroxide (H2O2) or staurosporine through activating a novel downstream p38 MAPK pathway. Intriguingly, after tPA treatment, resting (M0) macrophages and classically activated (M1) macrophages but not the alternative activated (M2) macrophages, are more resistant to apoptosis [34]. In obstruction-induced fibrotic kidneys, increased p90RSK activity associates with severer disease condition such as increased extracellular matrix (ECM) accumulation, enhanced M1 macrophage infiltration and more profound proinflammatory cytokine expression [34,42]. Thus, p90RSK promotes survival and accumulation of macrophages, especially the M1 macrophages, which produce a panoply of proinflammatory cytokines and chemokines, resulting in increased inflammatory cell recruitment, severer inflammatory response and excessive kidney injury.

### 3.3. p90RSK and Glomerular Diseases

Proteinuria is one of the common manifestations of glomerular diseases. In physiological condition, plasma is filtrated through glomerular basement membrane (GBM), resulting in urine containing largely the waste and toxins but not proteins larger than albumin. Podocytes cover the outer surface of the GBM. The long-interdigitated foot processes of podocytes form filtration slits and are critical for the integrity of glomerular filtration barrier. Podocytes are terminally differentiated cells and cannot regenerate when they are injured. In response to injury, such as puromycin aminonucleoside (PAN), podocytes undergo apoptosis and detach from GBM, leading to disruption of the integrity of glomerular filtration barrier and proteinuria [43]. Oh and colleagues found that calcimimetic R-568 induces ERK1/2-mediated p90RSK/CREB signalling cascade, alleviates PAN-induced proteinuria, attenuates glomerulosclerosis and improves glomerular filtration rate (GFR). They further clarified that p90RSK mediates protective effect of R568 through activating the pro-survival signalling of Bad and Bcl-xl and reducing PAN-induced podocyte apoptosis and damage [44].

### 3.4. p90RSK and Diabetic Nephropathy

Diabetic nephropathy is one of leading causes of end stage renal disease. Histologically, it is characterized by thickened tubular basal and glomerular membranes, excessive ECM deposition and progressive mesangial hypertrophy [45]. Mesangial cells constitute 30–40% of the total glomerular cell population and also contribute to ECM production [46]. Mesangial cell hypertrophy is often followed by matrix deposition and is associated with resultant glomerulosclerosis. TGF-β mediates the hypertrophic effect of hyperglycaemia [47]. Das and colleagues found that TGF-β induces ERK1/2 and p90RSK activation in mesangial cells. Activated p90RSK induces the phosphorylation and inactivation of eukaryotic elongation factor2 (eEF2) kinase, leading to decreased eEF2 phosphorylation and augmented eEF2 activity. eEF2 activation eventually induces protein synthesis and causes mesangial hypertrophy. Blocking the p90RSK signalling by using dominant-negative p90RSK inhibits TGF-β-stimulated mesangial hypertrophy [39].

### 3.5. p90RSK and Other Kidney Diseases

Emerging evidence also indicates that p90RSK involves in other kidney diseases. Zacchia and colleagues found that media acidification activates the citrate transporter NaDC-1 through Raf1, ERK1/2 and p90RSK signalling in the opossum kidney proximal tubule cells, suggesting that ERK1/2 and p90RSK may play a role in hypocitraturia and kidney stone formation [48]. Mizutani and colleagues found that severe acute respiratory syndrome (SARS)-coronavirus (CoV) infection induces p38 MAPK-mediated Ser380 phosphorylation of p90RSK but not through ERK1/2-induced Thr573 phosphorylation in kidney epithelial cells. This may be one of the underlying mechanisms of virus-induced kidney damage [49]. In the recovery of acute kidney injury, surviving epithelial cells de-differentiate, migrate to the injury site, proliferate and then re-differentiate to establish epithelial polarity and restore kidney function [50]. Tanimura and colleagues found that p90RSK is implied in the motility response of kidney epithelial cells induced by agents such as hepatocyte growth factor (HGF), phorbol 12-myristate 13-acetate (PMA) and epidermal growth factor (EGF) [51], indicating that p90RSK may mediate kidney regeneration and repair after acute injury.

## 4. p90RSK and Cardiovascular Disease

### 4.1. p90RSK and Atherosclerosis

Endothelial cells (ECs) are the single layer mesenchymal cells lining in the inner surface of blood vessels, lymphatics and other mesothelial-lined cavities. ECs not only separate all the tissue from circulating blood but also play an important role in many physiological functions such as controlling vascular tone, blood cell trafficking, immunity and haemostasis. ECs can sense and respond to the shear stress of blood flow, produce vasoactive factors, such as vasodilators and vasoconstrictors, procoagulants and anticoagulants, inflammatory and anti-inflammatory factors, fibrinolytic and antifibrinolytic factors, oxidizing and antioxidizing factors and other factors and help to maintain normal functions of blood vasculature. When exposed to various atherosclerosis risk factors, ECs express increased cell adhesion molecules, such as intercellular adhesion molecular-1 (ICAM-1), vascular cell adhesion molecular-1 (VCAM-1) and E-selectin, to enhance leukocyte recruitment, initiate inflammation and finally promote the progression of atherosclerosis [52,53].

Disturbed-flow (d-flow) is a known risk factor of atherosclerosis and activates p90RSK in ECs. Activated p90RSK phosphorylates T368 of sentrin/small ubiquitin-like modifier (SUMO)-specific protease 2 (SENP2), induces SENP2 nuclear export and reduces the SENP2 activity. SUMOylation is a post-translational modification in which a SUMO (small ubiquitin-like modifier) protein is conjugated to a target protein and regulates the localization, degradation, binding and activity of the target protein. SENPs family (SENP1-7) can convert precursor SUMO to mature SUMO and reverse the protein SUMOylation by de-SUMOylation of target protein. p90RSK activation-mediated nuclear export of SENP2 increases nuclear ERK5 and p53 SUMOylation, leading to EC apoptosis, inflammation and atherosclerotic plaque formation [53,54,55]. H2O2-induced EC p90RSK activation either associates with ERK 5 or directly phosphorylates S496 of ERK5. Both association with ERK5 and phosphorylation of S496 inhibit ERK5 transcriptional activity, which causes inhibition of KLF2 promoter activity, decrease of eNOS expression and increase of VCAM-1 expression, leading to atherosclerosis [56]. Vu and colleagues found that ionizing radiation induces p90RSK activation in ECs. Activated p90RSK increases NF-κB activation, VCAM-1 expression and EC apoptosis through phosphorylating ERK5 S496 and regulating its transcriptional activity. In vivo study confirmed that mice exposed to radiation exhibit higher endothelial VCAM-1 in the disturbed flow area. This finding may help to elucidate the underlying mechanisms of high incidence of cardiovascular disease in patients after radiation treatment [57].

Monocytes and macrophages are critical players in cardiovascular diseases and are involved in the process from atherosclerotic lesion formation and progression to cardiac regeneration after myocardial infarction [58]. Singh and colleagues found that p90RSK is activated by various combination antiretroviral therapies (cARTs) in monocytes and macrophages. Activated p90RSK phosphorylates S496 of ERK5, inhibits NRF2-ARE activity, which reduces the telomere length and decreases antioxidant expression, resulting in increased sensitivity of monocytes/macrophages to oxidative stress. Activated p90RSK in macrophages also induces the expression of pro-inflammatory genes such as TNFα and decreases the expression of efferocytosis-related genes, such as Gas6. These results were also validated in vivo using two different transgenic mouse models of atherosclerosis. Singh and colleagues found that WTp90rsk-MTg (over-expressing p90RSK in myeloid cells) mice have larger atherosclerotic lesion and necrotic core in the plaques than that from non-transgenic littermate control (NLC) mice. DNp90rsk-MTg (dominant-negative p90RSK in myeloid cells) mice exhibit smaller plaque with less developed necrotic core than that from NCL mice. Furthermore, monocytes from the peripheral whole blood of cART-treated HIV-positive patients display increased p90RSK activity and are more sensitive to reactive oxygen species (ROS), comparing to the monocytes from HIV-negative individuals [59]. Thus, p90RSK is a key player in regulating the formation and progression of atherosclerotic lesion through modulating efferocytosis, inflammation, antioxidant expression and senescence in monocytes and macrophages.

### 4.2. p90RSK and Myocardial Disease

p90RSK plays an important role in maintaining cardiac function and its aberrant activation contributes to the progression of myocardial hypertrophy and heart failure. Itoh and colleagues found that p90RSK mediates ROS and protein kinase C-β-induced cardiac troponin I phosphorylation [60]. p90RSK has been shown to be activated in pressure-overload-induced hypertrophic myocardium in guinea pigs and in the explanted hearts from patients with dilated cardiomyopathy [61]. Yamaguchi and colleagues showed that activated p90RSK reduces GSK-3β activity and contributes to the development of cardiac hypertrophy in mice expressing dysfunctional ryanodine receptor ion channel [62]. He and colleagues discovered that p90RSK activated by PGE2 in neonatal ventricular myocytes induces the expression of c-Fos, Egr-1 and BNP, leading to myocytes growth and probably cardiac hypertrophy [63]. p90RSK activation is also shown to mediate positive inotropic response to endothelin-1 in rat hearts [64] and stretch induces phosphorylation and activation of a serial of protein kinases including p90RSK in human myocardium and neonatal rat cardiac myocytes [65,66]. p90RSK also appears to play a role in cardiomyopathies after ischemia/reperfusion injury, since it has been reported that global ischemia/reperfusion induces rapid and transient activation of p90RSK in guinea pig hearts [67]. Jiang and colleagues found that p90RSK activation mediates the cardioprotective effect of protease activated receptor 2 (PAR2) in a rat heart ischemia/reperfusion model [68]. Itoh and colleagues studied the role of p90RSK in ischemic and diabetic myocardium using transgenic mice with cardiac-specific overexpression of wild-type p90 ribosomal S6 kinase (WT-p90RSK-Tg) and found that recovery of cardiac function after ischemia/reperfusion in isolated hearts from WT-p90RSK-Tg mice is significantly impaired in comparison with their wildtype controls [69]. Le and colleagues showed that activated p90RSK in diabetic hearts induces the phosphorylation of ERK5 S496, inhibits the association of ERK5 and CHIP ubiquitin ligase by binding to ERK5, which decreases the CHIP ubiquitin ligase activity, suppresses inducible cAMP early repressor (ICER) ubiquitination and degradation and finally promotes cardiac apoptosis [70]. These findings provide insights into the underlying mechanisms of high mortality and morbidity after myocardial infarction in diabetic patients.

### 4.3. P90RSK and Cardiac Arrhythmia

It has been found that activated p90RSK inhibits outward K^+^ channel activity, decreases currents such as fast transient outward K^+^ current (I(to,f)), slow delayed outward K^+^ current (I(K,slow)) and steady-state K+ current (I(SS)) in ventricular myocytes, prolongs the QT-interval and thus plays an important role in cardiac arrhythmia [71].

## 5. p90RSK and Cancer

p90RSK, as the downstream effector of the Ras/Raf/MEK/ERK signalling pathway, regulates numerous cell processes including cell growth, proliferation, survival and motility through phosphorylating different substrates and regulating various downstream signalling cascades. RSKs have been widely studied in cancer research including tumorigeneses, metastasis and chemoresistance.

In general, RSK1 and RSK2 are thought to promote many tumour progression through promoting proliferation and survival of cancer cells, while RSK3 and 4 may have some anti-tumour functions [2]. RSK1 and 2 promote cell growth through phosphorylating substrates, such as TSC2, raptor and eEF2 kinase, leading to the activation of mTOR pathway and increased translation of growth factors. RSK1 and 2 induce cell proliferation through phosphorylating substrates such as GSK3, c-Fos, Cdc25C and p27^kip1^, regulating the activity of cyclin-dependent kinases and increasing the protein level of cyclins. RSK1 and 2 promote cell survival through phosphorylating substrates such as CREB, Bad, Bim-EL and DAPK, decreasing the activity of pro-apoptotic molecules and inducing the transcription of pro-survival genes [2,72,73]. RSK1 and 2 also promote the invasion and metastasis of cancer cells from different tumours such as breast, colon, kidney, stomach and prostate, through mechanisms including (1) inducing a FRA1 and c-jun-dependent transcriptional program related to epithelial-mesenchymal transition (EMT); (2) phosphorylating SH3P2 and blocking its anti-motility effects; and (3) phosphorylating filamin A and suppressing the integrin activation through its increased binding activity [74]. In contrast, Lara and colleagues found that RSK1 has an anti-metastatic effect in lung cancer cells [73]. Thus, roles of RSKs in cancer are context-dependent and are far more complicated than our initial thoughts. Additionally, RSK1 and 2 play an important role in the chemoresistance that makes tumours more difficult to cure. Ray-David and colleagues found that RSK appears to contribute to the chemoresistance through phosphorylation of checkpoint kinase 1 (Chk1). RSK inhibition sensitizes melanoma cells to DNA-damaging agents by increasing Chk1 activity [75]. KRAS-mutant non-small cell lung cancer (NSCLC) is known to be resistant to general chemotherapy. Ganetespib, a Hsp90 inhibitor, is a promising therapy for NSCLC, however, its efficacy is limited by acquired resistance. Chatterjee and colleagues showed that activation of p90RSK/CDC25C pathway mediates the acquired resistance to Ganetespib through bypassing Ganetespib-induced G2-M cell cycle arrest [76].

## 6. Treatment Targeting p90RSK Signalling

Aberrant activation of p90RSK has been shown in numerous human diseases. Activated p90RSK phosphorylates various substrates and contributes to the initiation and development of numerous diseases through various signal pathways. As a common upstream mediator of various substrates, p90RSK inhibition provides a promising therapeutic strategy for numerous diseases. However, current options for specific inhibition of p90RSK remain very limited. Although it is presumable that inhibitors targeting upstream MEK1/2 or ERK1/2 also inhibit p90RSK activity, these inhibitors often cause more unwanted side effects than p90RSK-specific inhibitors [37].

SL0101, the first identified p90RSK-specific inhibitor, is isolated from the tropical plant *Forsteronia refracta*. However, application of SL0101 is limited because of its poor in vivo efficacy [2,72]. Ludwik and colleagues studied the effect of SL0101 analogues on the triple-negative breast cancer (TNBC) since around 85% of TNBC tumour samples show activated RSK. They found that a new SL0101 analogue, SL0101 (1b), specifically inhibits RSK1 and 2 in vitro and suppresses proliferation, survival and migration of TNBC cells. In vivo administration of SL0101 (1b), in the MCF-7 metastatic model, suppresses TNBC metastatic colonization with an efficiency comparable to FDA-approved MEK inhibitor, trametinib [77].

FMK-MEA, an RSK-selective and irreversible inhibitor, has been used in cardiovascular studies. In ECs, p90RSK activated by a combination of high glucose and low H2O2 inhibits the expression of KLF2 and eNOS and induces VCAM-1 expression. Whereas, incubation of ECs with FMK-MEA blocks these actions. Le and colleagues also checked the therapeutic effects of FMK-MEA in two diabetic animal models: STZ-induced diabetic mice and C57BL/6J-Ins2^Akita^ (Akita) mice. Expression and phosphorylation of p90RSK are increased in the aorta of diabetic mouse. These mice display increased leukocyte rolling and decreased Ach-induced vasodilation; and ECs isolated from diabetic mice showed increased expression of VCAM-1 and E-selectin and decreased expression of eNOS. However, after FMK-MEA treatment, dysfunctions of ECs and vasculatures are ameliorated [56]. In addition, FMK-MEA treatment has been shown to inhibit atherosclerotic plaque formation in an ApoE-/- atherosclerosis animal model [56]. These findings implicate that p90RSK inhibition is a promising therapeutic strategy for diabetes-mediated atherosclerosis.

BIX02565 is another confirmed inhibitor for RSK, at least RSK2. Shi and colleagues found that BIX02565 improves left ventricular pressure which is decreased by ischemia/reperfusion [2,78], suggesting that RSK inhibition may be a promising treatment for myocardial infarction.

BI-D1870 has been shown to inhibit p90RSK activation and block its effects in both in vivo and in vitro systems [34]. Takada and colleagues found that BI-D1870 administration protects mice from experimental autoimmune encephalomyelitis through reducing the infiltration of TH1 and TH17 cells into the CNS and decreasing mRNA level of Ccr6 in TH17 cells [79]. Thus, RSKs appear to be a promising therapeutic target for multiple sclerosis.

Notably, only a few p90RSK inhibition studies are carried out in animals, future studies regarding this aspect, as well as development of more efficient and specific p90RSK inhibitors, are warranted.

## 7. Conclusions

It is clear that p90RSK and other RSKs mediate various signal pathways to modulate diverse cellular processes and play critical roles in the pathogenesis and progression of human diseases. Of note, these effects are often context dependent and related to different initial receptors, downstream substrates and cell types. Challenges regarding p90RSK in disease pathophysiology, as well as development specific therapy targeting p90RSK, remain to be answered through future studies.
